# Multi-center evaluation of Neurophet AQUA for brain MRI segmentation: T1 compared with FreeSurfer and T2-FLAIR compared with ground truth

**DOI:** 10.3389/fneur.2025.1672133

**Published:** 2025-12-17

**Authors:** Hyunjae Yu, Hyunji Lee, Minho Lee, Donghyeon Kim, Lukas Pirpamer, Marco Duering, Sue Moy, Karl G. Helmer, Regina E. Y. Kim, for the Alzheimer's Disease Neuroimaging Initiative

**Affiliations:** 1 Research Institute, Neurophet Inc., Seoul, Republic of Korea; 2 Medical Image Analysis Center (MIAC), Basel, Switzerland; 3 Department of Biomedical Engineering, Translational Imaging in Neurology (ThINk) Basel, University of Basel, Basel, Switzerland; 4 Department of Radiology, Massachusetts General Hospital, Boston, MA, United States; 5 Harvard Medical School, Boston, MA, United States

**Keywords:** brain MRI segmentation, scan-rescan repeatability, inter-scanner reproducibility, white matter hyperintensity, automated segmentation, T1-weighted MRI, T2-FLAIR

## Abstract

**Introduction:**

Accurate segmentation of brain regions in magnetic resonance imaging (MRI) is essential for diagnosing and managing neurological diseases. FreeSurfer is a widely used tool for brain MRI segmentation, but its limitations in speed and usability pose challenges in clinical practice. Neurophet AQUA, an advanced automated segmentation tool, aims to overcome these challenges by offering rapid and reliable segmentation. This study evaluates two segmentation pipelines: (1) a T1-based brain region segmentation pipeline, comparing the performance and reliability of Neurophet AQUA and FreeSurfer v7.3.2 using the standard recon-all pipeline in segmenting gray matter, white matter, and subcortical structures; and (2) a T2-FLAIR-based white matter lesion segmentation pipeline of Neurophet AQUA, assessing the detection of white matter hyperintensities (WMH).

**Methods:**

Four main datasets were used. For the T1-based segmentation pipeline, the Alzheimer’s Disease Neuroimaging Initiative (ADNI) dataset was used to compare the segmentation results of Neurophet AQUA and FreeSurfer, with quality assessed by expert evaluation. The MarkVCID dataset was used to evaluate the scan-rescan repeatability and inter-scanner reproducibility of Neurophet AQUA. For the T2-FLAIR-based pipeline, WMH segmentation performance was assessed using 2D and 3D FLAIR sequences from the ADNI dataset by comparing the segmentations to ground truth (GT) labels and calculating Dice similarity coefficients (DSC).

**Results:**

Segmentation quality and reliability showed that Neurophet AQUA and FreeSurfer achieved comparable performance in most regions, with no significant differences. However, Neurophet AQUA had significantly faster processing time. In intracranial volume (ICV) measurements, Neurophet AQUA showed better repeatability than FreeSurfer in both rescans (ICC: 0.999 vs. 0.991) and inter-scanner settings (ICC: 0.983 vs. 0.866). AQUA also demonstrated consistent WMH segmentation across 2D and 3D FLAIR images.

**Conclusion:**

Neurophet AQUA demonstrated high segmentation accuracy and excellent repeatability in rescanned measurements, as well as exploratory evidence of inter-scanner reproducibility on T1-weighted MRI, showing comparable performance to established tools such as FreeSurfer. It also showed consistent WMH segmentation across FLAIR types. Neurophet AQUA is highly suitable for clinical applications that require accurate analysis, high repeatability and reproducibility, and rapid brain MRI processing, making it particularly well-suited for multicenter research studies.

## 1 Introduction

Accurate volumetric measurements of brain regions are essential for tracking neurodegenerative diseases. Various automated brain segmentation tools have been developed, including widely used research software such as FreeSurfer (1), FSL-FIRST (2), ANTs (3), and SPM (4), as well as newer deep-learning-based solutions like FastSurfer (5) and the free VolBrain platform, which does not require local installation (6). These tools differ in their segmentation methodologies, computational efficiency, and clinical applicability.

Neurophet AQUA is a recently developed segmentation tool designed for efficient and reliable analysis of structural brain changes, particularly in the context of neurodegenerative diseases. It is FDA-approved for clinical use (7). The tool provides rapid processing while retaining the comprehensive segmentation capabilities typical of established neuroimaging pipelines. For instance, a recent study by Lee et al. (8) demonstrated that while FreeSurfer typically requires approximately 1 h per image, Neurophet AQUA completes the analysis in under 5 min on average. Moreover, Neurophet AQUA incorporates a deep-learning-based T2-FLAIR segmentation algorithm for fully automated identification of white matter hyperintensities (WMHs) in T2-FLAIR scans, showing particularly strong performance in segmenting small-sized WMHs (9, 10).

While the segmentation capabilities of Neurophet AQUA have previously been documented and positively evaluated (9), there remains a need for objective performance comparisons to establish its reliability in a broader context. In this study we aimed to benchmark Neurophet AQUA against FreeSurfer, a well-established and widely open source used tool, validated across diverse populations and MRI scanners (11), using expert radiologist assessments of T1-weighted MRI scans. We further validate the technical reliability of both tools through scan-rescan repeatability to determine consistency in results when images are acquired twice on the same MRI device (12–14). Additionally, we evaluate inter-scanner reproducibility to assess whether consistent results are obtained when the same patient is scanned on different MRI machines (15).

In this study, we aimed to benchmark Neurophet AQUA against FreeSurfer for T1-weighted brain segmentation, and to evaluate its scan-rescan repeatability and inter-scanner reproducibility across multi-center MRI data. Additionally, we assessed AQUA’s T2-FLAIR-based WMH segmentation against ground truth labels. This comprehensive evaluation was designed to establish AQUA’s reliability and clinical applicability as a rapid, automated segmentation tool.

## 2 Method

### 2.1 Datasets

**Figure 1:**
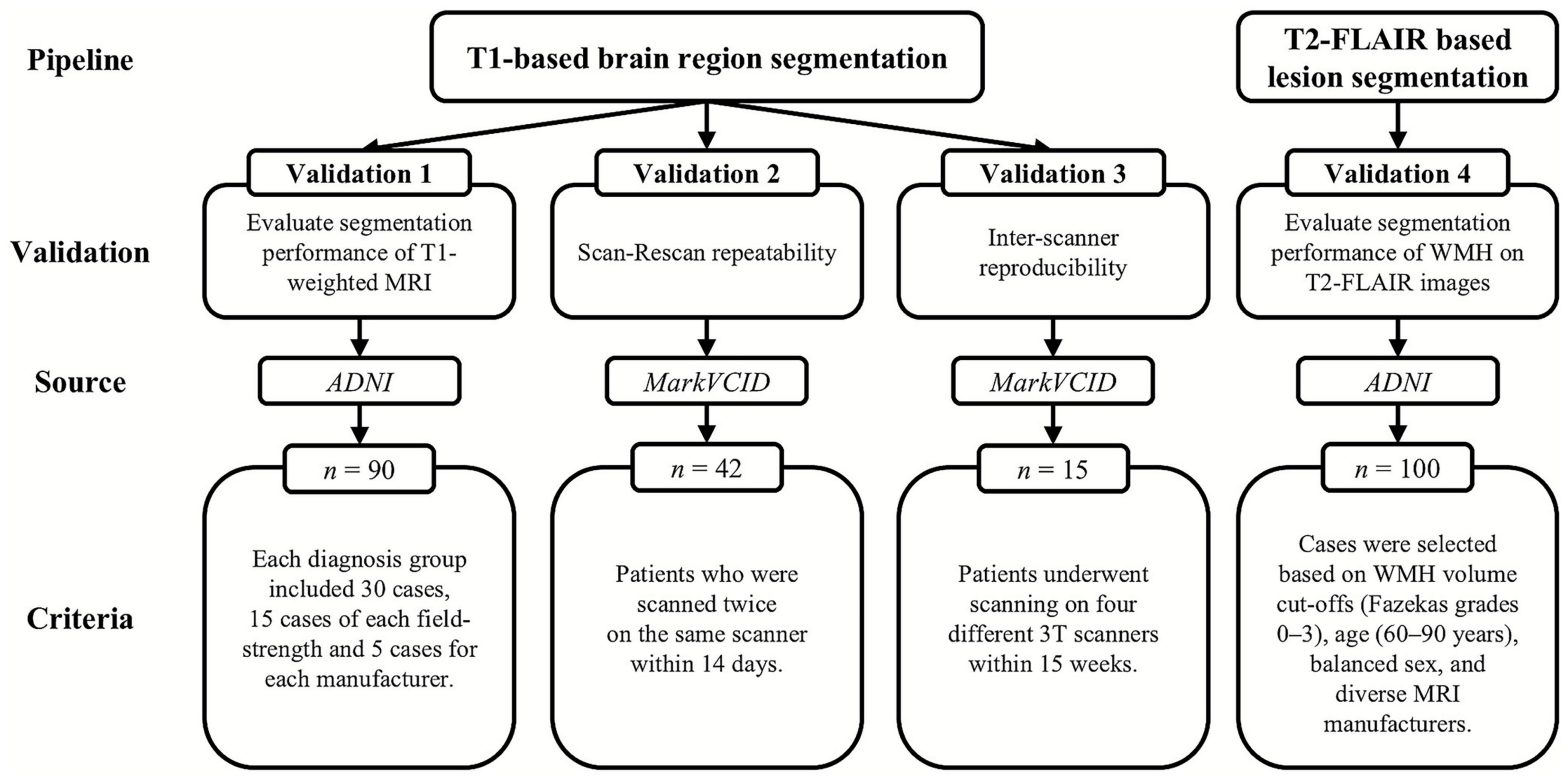
Flow chart. The flow chart illustrates the study design and participant inclusion process. It shows the selection of participants from the initial screening through to the final analysis, detailing the number of subjects excluded at each stage. The chart includes steps such as inclusion criteria, scanning procedures, and segmentation analysis, providing a visual overview of the study methodology. ADNI, Alzheimer’s Disease Neuroimaging Initiative.

To evaluate Neurophet AQUA’s performance, four validation datasets were used, selected from the Alzheimer’s Disease Neuroimaging Initiative (ADNI) (16, 17) and the MarkVCID consortium (18–20) (Figure 1). The ADNI data used in the preparation of this article were obtained from the Alzheimer’s Disease Neuroimaging Initiative (ADNI) database (adni.loni.usc.edu). The ADNI was launched in 2003 as a public-private partnership, led by Principal Investigator Michael W. Weiner, MD. The primary goal of ADNI has been to test whether serial magnetic resonance imaging (MRI), positron emission tomography (PET), other biological markers, and clinical and neuropsychological assessment can be combined to measure the progression of mild cognitive impairment (MCI) and early Alzheimer’s disease (AD).

**Table 1:** Demographic and imaging characteristics of validation dataset 1 used for T1-weighted MRI segmentation performance evaluation.

Variables	CN	MCI	ADD	*p*-value^†^
	(*N* = 30)	(*N* = 30)	(*N* = 30)	
Sex - Female	18 (60.0%)	18 (60.0%)	18 (60.0%)	NS
Age (Mean ± SD)	74.1 ± 8.2	72.9 ± 8.8	73.2 ± 9.3	NS
Field strength				NS
1.5 T	15 (50.0%)	15 (50.0%)	15 (50.0%)	
3 T	15 (50.0%)	15 (50.0%)	15 (50.0%)	
Manufacturer				NS
Siemens	10 (33.3%)	10 (33.3%)	10 (33.3%)	
Philips	10 (33.3%)	10 (33.3%)	10 (33.3%)	
GE	10 (33.3%)	10 (33.3%)	10 (33.3%)	

^†^The continuous variable was analyzed using one-way ANOVA, while Chi-square tests were conducted for categorical variables. A *p*-value ≥ 0.05 indicates no significant difference between groups.

CN, cognitive normal; MCI, mild cognitive impairment; ADD, Alzheimer’s disease dementia; GE, general electronics; SD, standard deviation; NS, not significant.

First, for T1-based segmentation evaluation, 90 ADNI cases (Validation dataset 1) were categorized as cognitively normal (CN), mild cognitive impairment (MCI), or Alzheimer’s disease dementia (ADD), with 30 subjects per group. Each group included equal numbers of 1.5 T and 3 T MRI scans, evenly distributed across Siemens, Philips, and General Electric (GE). Sex and age were also matched to minimize intergroup differences (Table 1).

**Table 2:** Demographic and MRI characteristics of validation dataset 4 (ADNI Subset) stratified by 2D and 3D T2-FLAIR sequences for WMH segmentation evaluation.

Variables	2D FLAIR	3D FLAIR	*p*-value^†^
	(*N* = 40)	(*N* = 60)	
Sex - Female	19 (47.5%)	27 (45.0%)	NS
Age (Mean ± SD)	75.0 ± 7.7	79.0 ± 6.9	0.007
WMH Group			NS
WMH score 0	10 (25.0%)	15 (25.0%)	
WMH score 1	10 (25.0%)	15 (25.0%)	
WMH score 2	10 (25.0%)	15 (25.0%)	
WMH score 3	10 (25.0%)	15 (25.0%)	
Manufacturer			NS
Siemens	18 (45.0%)	26 (43.3%)	
Phillips	13 (32.5%)	20 (33.3%)	
GE	9 (22.5%)	14 (23.3%)	

^†^The continuous variable was analyzed using independent two-sample t-test, while Chi-square test were conducted for categorical variables. A *p*-value ≥ 0.05 indicates no significant difference between groups.

WMH, white matter hyperintensity; GE, general electronics; NS, not significant.

Second, for scan-rescan repeatability (Validation dataset 2), we analyzed 42 MarkVCID patients who underwent two MRI scans on the same scanner within 14 days. Inter-scanner reproducibility (Validation dataset 3) was assessed using 15 patients scanned on four different 3 T MRI scanners (Siemens Trio, Siemens Prisma, GE Discovery 750, and Philips Achieva) within 15 weeks.

Third, for T2-FLAIR-based lesion segmentation evaluation, 100 ADNI cases (Validation dataset 4) were selected using WMH volume cut-offs based on Fazekas grades (0–3). Instead of visual grading, pre-defined volumetric thresholds from previous literature (21) were applied (WMH score 0: ≤1 mL; score 1: 1–7 mL; score 2: 7–20 mL; score 3: >20 mL, approximately corresponding to Fazekas grades 0–3). Subjects aged 60–90 were evenly distributed across Siemens, Philips, and GE scanners (Table 2). Cases with stroke, metastasis, hemorrhage, or traumatic brain injury were excluded, along with scans having severe artifacts, low signal-to-noise ratio (SNR), or unclear lesion boundaries.

Tables 1, 2 summarize the demographic and MRI characteristics of Validation datasets 1 and 4, respectively.

### 2.2 Segmentation methods

#### 2.2.1 Neurophet AQUA

The Neurophet AQUA T1-weighted MRI segmentation algorithm was trained on multi-center and multi-vendor data (8). The training set consisted of 746 T1-weighted images acquired with Philips, Siemens, and GE MRI scanners: 300 high-quality, 363 medium-quality, and 83 low-quality. All training data included manually annotated ground truth labels, performed by certified radiologists. This deep learning-based algorithm uses the SAU-Net architecture, based on Nested U-Net (8, 22).

Preprocessing included the right anterior–posterior orientation shift and 1 mm isotropic resampling. Histogram-based intensity normalization was applied before dividing the images into local patches, which were used to iteratively train the network with data augmentation techniques. High-quality data was trained using a 3D ResNeSt block with a per-channel attention module and EvoNorm layer (23, 24). Hierarchical transfer learning was performed on medium- and low-quality data. The final segmentation, completed in about 5 min, covered over 100 regions of interest in the cortex and subcortex (8).

The Neurophet AQUA T2-FLAIR segmentation algorithm was trained on 239 FLAIR images, consisting of both 2D and 3D FLAIR sequences, from a multi-site dataset across four Korean hospitals. Manual lesion labeling (0 = non-WMH, 1 = WMH) was performed by three board-certified radiologists (9).

Preprocessing involved resampling MRIs to 1 mm^2^ isotropic space and cranial stripping using HD-BET (25). Data augmentation was applied using TorchIO technology.^1^ The model, based on 2D U-Net and patch-based training, incorporated a bottleneck attention module to improve small WMH performance. A unique training process is sorted datasets by ground truth data volume. The final model uses an ensemble method, merging results from models with and without histogram normalization, leading to enhanced segmentation performance, particularly for small WMHs (9, 10).

Neurophet AQUA, a commercial software, was developed with the participation of authors Donghyeon Kim and Minho Lee. The installation requirements for Neurophet AQUA are Windows 10 (64-bit), an Intel i7-8700 CPU (6 cores, 3.2 GHz), 32 GB or more of RAM, a 256 GB or larger SSD and a 4 TB or larger HDD for storage, and an NVIDIA GeForce RTX 3060 GPU with 12 GB or more of memory, CUDA 11.0, and NVIDIA driver version 455 or later.

#### 2.2.2 FreeSurfer

We used the recon-all cross-sectional pipeline from FreeSurfer version 7.3.2^2^ with default settings. This pipeline includes several stages, such as cortical surface extraction, subcortical segmentation, and spatial normalization, enabling comprehensive structural analysis of T1-weighted MR images (1). Additionally, cortical lobes and hippocampal masks were extracted from the aparc+aseg segmentation by merging labels according to the defined structures (26). The entire pipeline takes approximately 2 h to complete.

### 2.3 Performance assessment

The performance of Neurophet AQUA was validated in three key areas: (1) segmentation quality of cortical and subcortical regions, (2) repeatability and reproducibility of volumetric measurements (technical validation), and (3) the accuracy of WMH segmentation. For T1-weighted images, (1) and (2) were evaluated by benchmarking against FreeSurfer. For T2-FLAIR scans, (3) was assessed using ground truth-based Dice similarity coefficient (DSC) evaluation. All quantitative analyses (Validations 1–4) were independently performed by the Medical Image Analysis Center (MIAC AG, Basel, Switzerland), which was not involved in the software development.

#### 2.3.1 Segmentation quality assessment

Segmentation quality was evaluated based on the accuracy of cortical gray matter (GM)–white matter (WM) boundaries and the localization accuracy of key brain regions, including the frontal, parietal, temporal, and occipital lobes, as well as the hippocampus. Quality was rated on a three-point ordinal scale (1 = poor, 2 = medium, 3 = ideal) and compared between Neurophet AQUA and FreeSurfer. Additionally, overall segmentation quality was directly compared at the patient level, without distinguishing specific brain regions. All quality assessments were conducted by blinded raters from the MIAC AG analysis team, who were trained experts following the standard operating procedures of MIAC AG and blinded to the identity of the segmentation algorithm. Detailed expert evaluations of segmentation quality, categorized into cortical tissue boundaries, lobar segmentation, and hippocampal segmentation, are provided in Supplementary Tables S1, S2. Additionally, the example images are presented in Supplementary Figures S1, S2.

#### 2.3.2 Technical validation

For technical validation, we assessed both scan-rescan repeatability and inter-scanner reproducibility. Repeatability was evaluated by measuring brain volumes from two T1-weighted MRI scans acquired on the same scanner within a 14-day interval. Reproducibility was assessed by analyzing brain volumes from four T1-weighted MRI scans obtained using four different 3 T scanners across multiple sites within a 15-week period. The evaluated volumetric measures included total brain volume, intracranial cavity volume (ICV), and the cortical volumes of the frontal, parietal, temporal, and occipital lobes, as well as hippocampal volume.

#### 2.3.3 WMH segmentation performance

WMH segmentation performance was assessed using the Dice Similarity Coefficient (DSC) (27). Neurophet AQUA outputs were compared against manually annotated ground truth WMH masks, labeled by expert radiologists following the STRIVE-2 criteria (28).

### 2.4 Statistical analysis

Demographic and imaging characteristics of the datasets used for T1-weighted MRI segmentation quality assessment and T2-FLAIR segmentation performance evaluation were analyzed. For continuous variables such as age, one-way ANOVA or independent t-tests were performed, while categorical variables (e.g., gender, field strength) were analyzed using chi-square tests.

Segmentation quality ratings between Neurophet AQUA and FreeSurfer were compared using the Wilcoxon signed-rank test (29, 30). Additionally, segmentation differences at the patient level, considering recognition type, field strength, and scanner manufacturer, were assessed using chi-square tests.

For scan-rescan volume differences, normality was assessed using the Shapiro–Wilk test. If the data were normally distributed, paired t-tests were conducted; otherwise, the Wilcoxon signed-rank test was applied. Scan-rescan repeatability and inter-scanner reproducibility were evaluated using the intraclass correlation coefficient (ICC) with a two-way random-effects model for absolute agreement. The ICC values were interpreted as follows: ICC < 0.5 (poor), 0.5 ≤ ICC < 0.75 (moderate), 0.75 ≤ ICC < 0.9 (good), and ICC ≥ 0.9 (excellent) (31).

To assess bias and agreement in segmented volumes, Bland–Altman plots were utilized for both scan-rescan repeatability and inter-scanner reproducibility. Limits of Agreement (LOA) were calculated as 1.96 times the standard deviation of volume differences, while the Limits of Agreement for Multiple measurements (LOAM) were applied to account for inter-scanner variability (32, 33).

For WMH segmentation, DSC differences between 2D and 3D FLAIR sequences were analyzed. The Shapiro–Wilk test was used to assess normality, and variance homogeneity was tested using Levene’s test. If variance homogeneity was not met, a Welch’s two-sample t-test was performed; otherwise, either a standard two-sample t-test or a Mann–Whitney U test was applied, depending on data distribution.

All statistical analyses were performed using R version 4.4.0. Two-tailed tests were applied, and statistical significance was set at *p* < 0.05.

## 3 Results

### 3.1 Segmentation quality of T1-weighted MRI segmentation

The segmentation quality ratings for GM–WM boundaries comparing Neurophet AQUA and FreeSurfer are summarized in Table 3 for the four lobes (frontal, temporal, parietal, occipital) and the hippocampus. Among these, only the left hippocampus showed significantly higher quality with Neurophet AQUA (2.83 ± 0.37 vs. 2.67 ± 0.54, *p* = 0.029). Although no significant differences were found in other regions, Neurophet AQUA received slightly higher ratings in five regions and slightly lower in one. However, after applying Bonferroni correction for multiple comparisons (adjusted *α* = 0.005), all regional differences, including those in the left hippocampus, were no longer statistically significant.

**Table 3:** Comparison of segmentation quality scores between Neurophet AQUA and FreeSurfer on T1-weighted MRI from validation dataset 1.

Regions		Mean (Number of subjects per scores of 1/2/3)		*p*-value^†^
		Neurophet AQUA	FreeSurfer	
Cortical GM-WM boundaries		2.40 (13/28/49)	2.43 (12/27/51)	NS
Frontal lobe	Left	3.00 (0/0/90)	2.98 (1/0/89)	NS
	Right	3.00 (0/0/90)	3.00 (0/0/90)	na
Temporal lobe	Left	3.00 (0/0/90)	3.00 (0/0/90)	na
	Right	2.99 (0/1/89)	2.99 (0/1/89)	NS
Parietal lobe	Left	2.94 (0/5/85)	2.93 (1/4/85)	NS
	Right	2.99 (0/1/89)	2.94 (0/5/85)	NS
Occipital lobe	Left	2.94 (0/5/85)	2.96 (0/4/86)	NS
	Right	2.98 (0/2/88)	2.93 (0/6/84)	NS
Hippocampus	Left	2.83 (0/15/75)	2.67 (3/24/63)	0.029
	Right	2.81 (1/15/74)	2.77 (0/21/69)	NS

^†^The Wilcoxon signed-rank test was performed. A *p*-value ≥ 0.05 indicates no significant difference between Neurophet AQUA and FreeSurfer.

GM, gray matter; WH, white matter; Left, left hemisphere; Right, right hemisphere; na, not applicable; NS, not significant.

**Figure 2:**
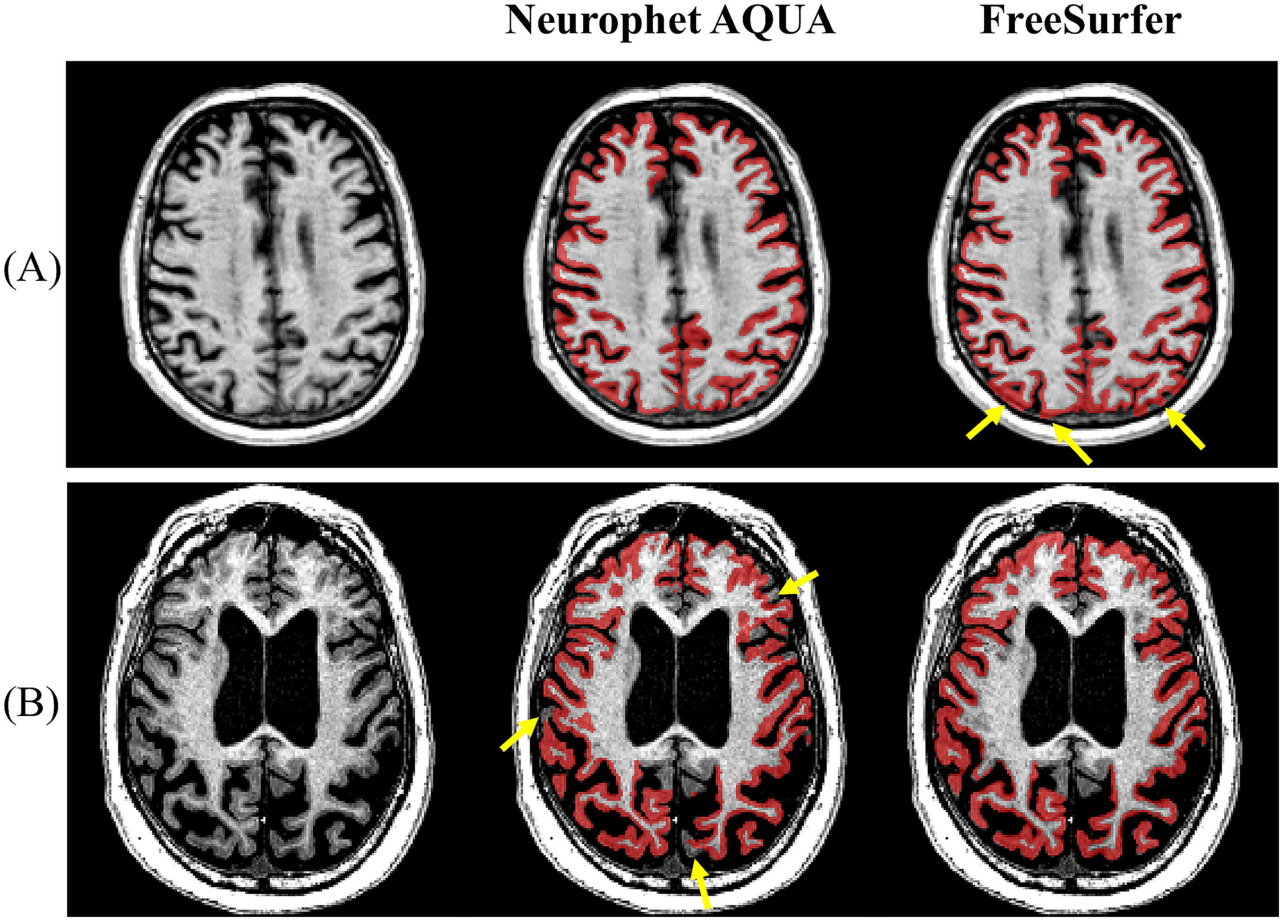
Representative examples of GM–WM–CSF boundary delineation by Neurophet AQUA and FreeSurfer. **(A)** Neurophet AQUA provided clearer separation of gray matter, white matter, and cerebrospinal fluid boundaries. **(B)** FreeSurfer provided sharper boundary separation in another case.

**Figure 3:**
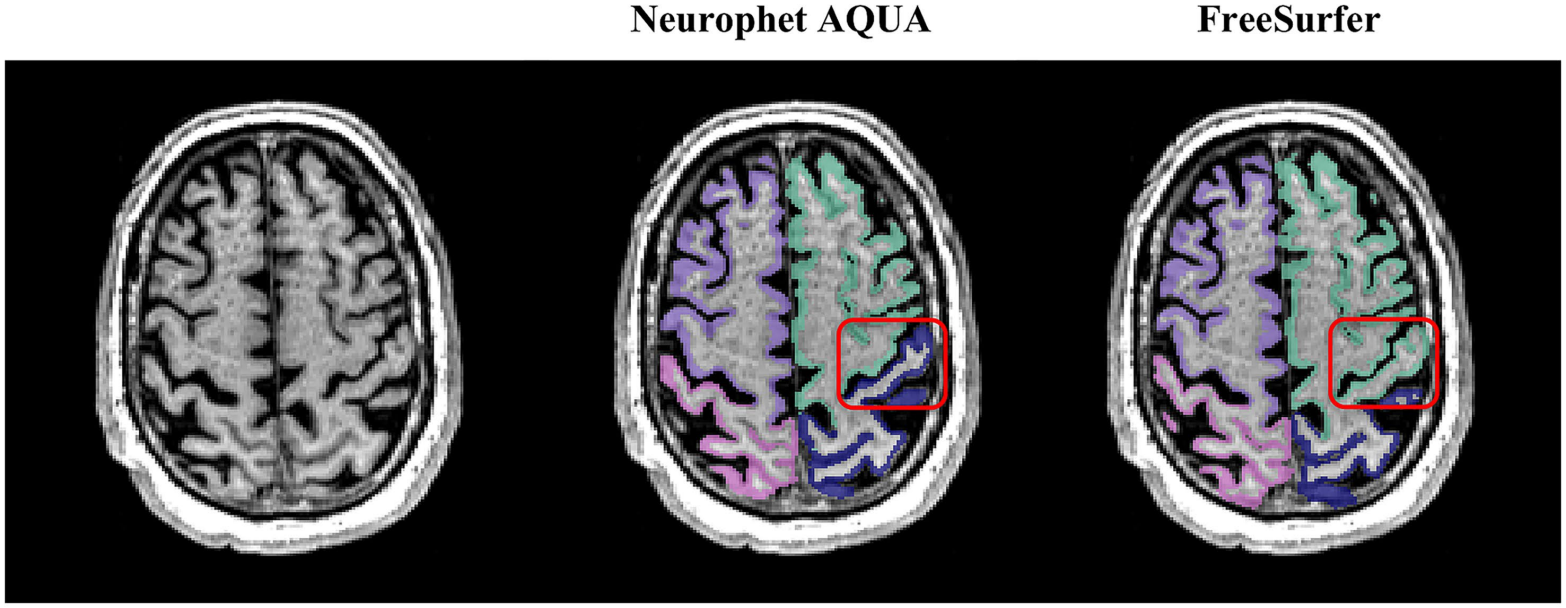
Representative example of lobe boundary detection. Neurophet AQUA demonstrated more precise delineation of lobe boundaries.

To more clearly illustrate segmentation quality, representative visual examples are presented in Figures 2, 3. Specifically, Figure 2A shows a case where Neurophet AQUA more clearly distinguished the gray matter-white matter-cerebrospinal fluid boundary, while Figure 2B shows a case where FreeSurfer performed a more distinct boundary separation. Furthermore, as seen in Figure 3, Neurophet AQUA demonstrated superior performance in detecting lobar boundaries, and no instances were observed where FreeSurfer outperformed AQUA in this aspect. A visual comparison of segmentation results between the two tools is shown in Table 4. Among 90 subjects, segmentation quality was rated equal in 55 (61.1%), better for Neurophet AQUA in 17 (18.9%), and better for FreeSurfer in 18 (20.0%). Overall, segmentation quality was comparable, with most subjects receiving identical scores.

**Table 4:** Visual comparison of T1-weighted MRI segmentation performance between Neurophet AQUA and FreeSurfer.

Variables	Neurophet AQUA better (*N* = 17)	FreeSurfer better (*N* = 18)	*p-value* ^†^	Equal performance (*N* = 55)
Cognitive type			NS	
CN	2 (11.8%)	5 (27.8%)		23 (41.8%)
MCI	8 (47.1%)	8 (44.4%)		14 (25.5%)
ADD	7 (41.2%)	5 (27.8%)		18 (32.7%)
Field strength			NS	
1.5 T	6 (35.3%)	6 (33.3%)		33 (60.0%)
3 T	11 (64.7%)	12 (66.7%)		22 (40.0%)
Manufacturer			NS	
Siemens	7 (41.2%)	7 (38.9%)		16 (29.1%)
Philips	5 (29.4%)	3 (16.7%)		22 (40.0%)
GE	5 (29.4%)	8 (44.4%)		17 (30.9%)

^†^The Chi-square test was performed. A *p*-value ≥ 0.05 indicates no significant difference between groups.

SD, standard deviation; CN, cognitive normal; MCI, mild cognitive impairment; ADD, Alzheimer’s disease dementia; GE, general electronics; NS, not significant.

Among the 35 subjects showing differences, we examined the influence of cognitive type, field strength, and scanner manufacturer. For cognitive type, both tools had the highest number in the MCI group, with no significant difference. Both tools also had more 3 T than 1.5 T cases, with no difference in performance based on field strength. Regarding manufacturing, 7 of the 17 better-rated Neurophet AQUA cases were from Siemens, while 8 of the 18 better FreeSurfer cases were from GE. Again, no significant difference was observed based on manufacturer.

### 3.2 Scan-rescan repeatability

The scan-rescan repeatability results for Neurophet AQUA and FreeSurfer are summarized in Table 5. First, regarding volume differences between scan and rescan images, Neurophet AQUA showed no significant bias in any region, including the whole brain, ICV, frontal lobe, temporal lobe, parietal lobe, occipital lobe, and hippocampus. In contrast, FreeSurfer exhibited a significant volume difference in ICV (1500.0 ± 160.2 vs. 1509.3 ± 153.8, *p* = 0.004), whereas no significant differences were observed in other regions.

**Table 5:** Comparison of scan–rescan repeatability for volumetric measurements between Neurophet AQUA and FreeSurfer using T1-weighted MRI from validation dataset 2.

Regions		Neurophet AQUA			FreeSurfer			ICC	
		Test Mean (SD)	Retest Mean (SD)	*p*-value^†^	Test Mean (SD)	Retest Mean (SD)	*p*-value^†^	AQUA	FS
ICV		1493.4 (112.3)	1493.4 (111.0)	NS	1500.0 (160.2)	1509.3 (153.8)	0.004	0.999	0.991
Whole brain		1031.6 (76.7)	1032.5 (74.4)	NS	1067.8 (114.9)	1065.3 (107.8)	NS	0.993	0.986
Frontal lobe	Left	74.0 (8.2)	73.6 (9.2)	NS	73.0 (13.7)	73.1 (13.2)	NS	0.953	0.987
	Right	74.4 (8.3)	74.0 (9.4)	NS	73.6 (13.4)	73.4 (12.9)	NS	0.940	0.982
Temporal lobe	Left	49.7 (5.1)	49.3 (5.8)	NS	44.1 (9.8)	44.6 (9.0)	NS	0.947	0.964
	Right	48.0 (4.6)	47.7 (5.5)	NS	43.7 (9.6)	44.2 (9.1)	NS	0.925	0.962
Parietal lobe	Left	49.4 (4.9)	49.1 (5.5)	NS	46.5 (8.5)	46.6 (8.3)	NS	0.920	0.971
	Right	50.5 (5.3)	50.2 (5.9)	NS	47.2 (9.7)	47.4 (9.3)	NS	0.937	0.962
Occipital lobe	Left	21.1 (2.7)	21.0 (3.2)	NS	20.0 (3.9)	20.0 (3.8)	NS	0.844	0.910
	Right	22.3 (2.9)	21.9 (3.6)	NS	20.9 (4.3)	21.1 (4.4)	NS	0.814	0.843
Hippocampus	Left	3.4 (0.4)	3.4 (0.4)	NS	3.5 (0.5)	3.5 (0.4)	NS	0.883	0.876
	Right	3.4 (0.4)	3.5 (0.4)	NS	3.6 (0.5)	3.6 (0.5)	NS	0.821	0.971

Volume measurements for each region were in cc.

^†^The paired t-test was performed on normally distributed data. For continuous variables that did not show normal distributions, the Wilcoxon signed rank test was performed. A *p*-value ≥ 0.05 indicates no significant difference between test and retest.

ICC, intraclass correlation coefficient; FS, FreeSurfer; ICV, intracranial volume; Left, left hemisphere; Right, right hemisphere; SD, standard deviation; NS, not significant.

Second, ICC values for both Neurophet AQUA and FreeSurfer were above 0.8, indicating good to excellent repeatability (ICC: Neurophet AQUA = 0.814–0.999, FreeSurfer = 0.843–0.991). Neurophet AQUA demonstrated excellent repeatability (ICC ≥ 0.9) for ICV, as well as the frontal, temporal, and parietal lobes, while the occipital lobe and hippocampus exhibited good repeatability (ICC > 0.8). Similarly, FreeSurfer showed excellent repeatability (ICC ≥ 0.9) in the frontal, temporal, parietal, left occipital, and right hippocampus, whereas the right occipital lobe and left hippocampus displayed good repeatability with ICCs above 0.8.

**Figure 4:**
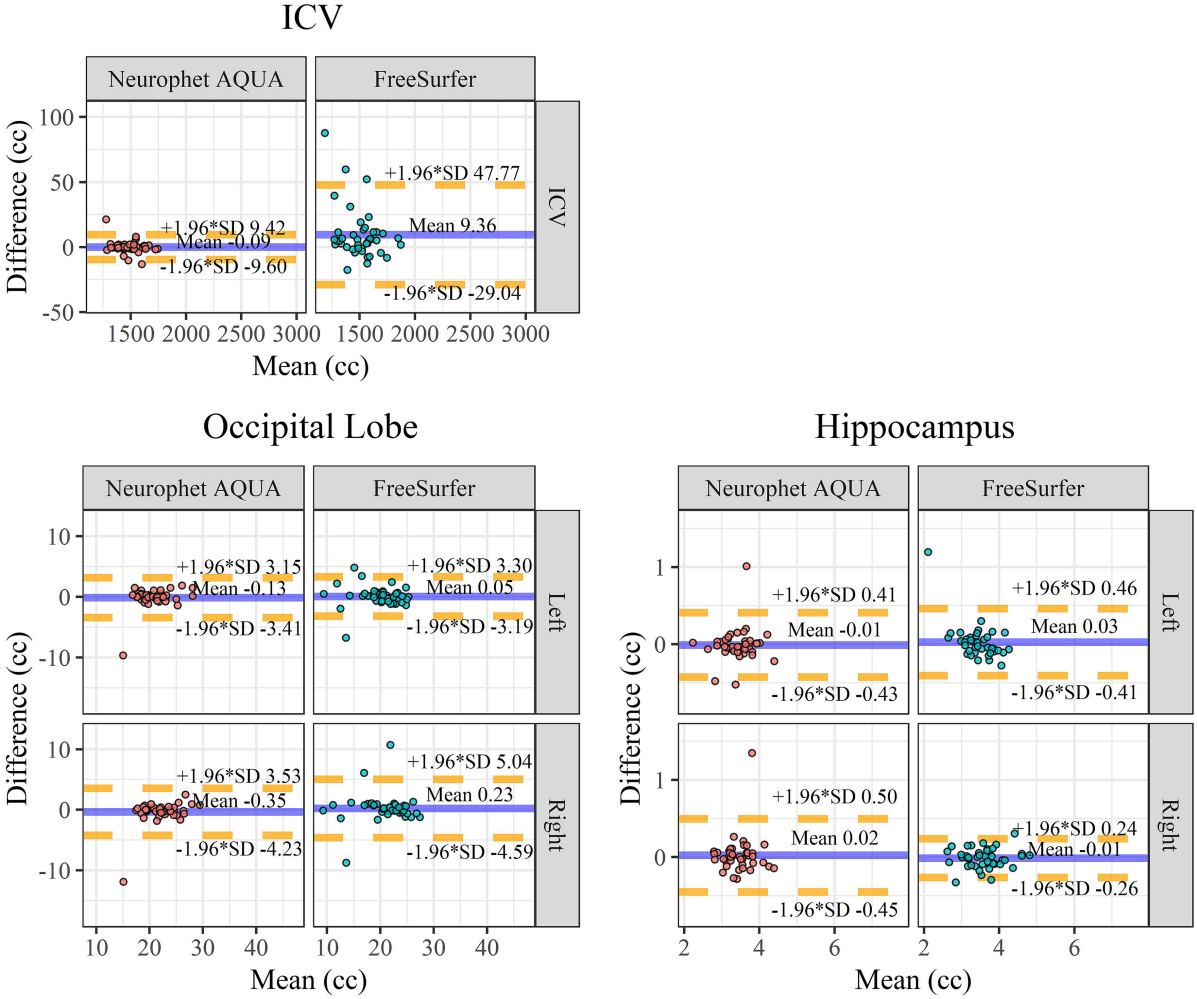
Bland–Altman plot for scan-rescan repeatability. Bland–Altman plots for scan-rescan repeatability comparing ICV, occipital lobe, and hippocampus between Neurophet AQUA and FreeSurfer. *X*-axis: mean brain volume between the scan and rescan. The unit of volume on the *X*-axis is cc. *Y*-axis: volume difference between the scan and rescan. The mean of difference is indicated by the blue line. The lower (−1.96 SD) and upper (+1.96 SD) limits of agreement are indicated by dotted yellow lines. A negative difference on the *y*-axis indicates that the volume measured in the retest image was smaller than the volume measured in the scan image. ICV, intracranial volume; Left, left hemisphere; Right, right hemisphere; SD, standard deviation.

The results of the Bland–Altman analysis are shown in Figure 4. For ICV, FreeSurfer exhibited larger scan-rescan differences compared to Neurophet AQUA. In the occipital lobes, although ICC values for Neurophet AQUA (left: 0.844, right: 0.814) were lower than those for FreeSurfer (left: 0.910, right: 0.843), Neurophet AQUA had fewer outliers. In the hippocampus, Neurophet AQUA had more outliers in the left hemisphere, but its 95% LoA values were comparable to those of FreeSurfer. In the right hemisphere, Neurophet AQUA’s 95% LoA covered almost twice the range of FreeSurfer’s; however, most data points were concentrated within a similar range, except for one outlier. A summary of the Bland–Altman analysis results for all regions is provided in Supplementary Table S3 and Supplementary Figure S3.

### 3.3 Inter-scanner reproducibility

The inter-scanner reproducibility of Neurophet AQUA and FreeSurfer was evaluated and is summarized in Table 6. Across all analyzed regions, both methods demonstrated at least moderate agreement (ICC: Neurophet AQUA = 0.715–0.983, FreeSurfer = 0.650–0.882). The average ICC for ICV, whole brain, and the four lobes (frontal, temporal, parietal, occipital) as well as the hippocampus was higher for Neurophet AQUA (average ICC = 0.850) compared to FreeSurfer (average ICC = 0.788), though both methods exhibited good agreement.

**Table 6:** Inter-scanner reproducibility comparison of segmentation results between Neurophet AQUA and FreeSurfer using T1-weighted MRI from validation dataset 3.

Regions		ICC	
		Neurophet AQUA	FreeSurfer
ICV		0.983	0.866
Whole brain		0.935	0.882
Frontal lobe	Left	0.789	0.805
	Right	0.796	0.669
Temporal lobe	Left	0.875	0.791
	Right	0.873	0.741
Parietal lobe	Left	0.869	0.813
	Right	0.854	0.730
Occipital lobe	Left	0.838	0.864
	Right	0.864	0.650
Hippocampus	Left	0.813	0.782
	Right	0.715	0.864
Average		0.850	0.788

ICC, interclass correlation coefficient; ICV, intracranial volume; Left, left hemisphere; Right, right hemisphere.

Neurophet AQUA achieved excellent agreement for ICV (ICC = 0.983) and brain volume (ICC = 0.935), while demonstrating moderate consistency in the right hippocampus (ICC = 0.715). All other regions showed good agreement. In contrast, FreeSurfer displayed good agreement in most regions but only moderate agreement in the right frontal, temporal, parietal, and occipital lobes (ICC = 0.650–0.741).

The agreement between scanner-derived volumes was further analyzed using the LOAM method. The results, along with visualizations, are presented in Supplementary Table S4 and Supplementary Figure S4.

### 3.4 WMH segmentation by T2-FLAIR sequence

**Figure 5:**
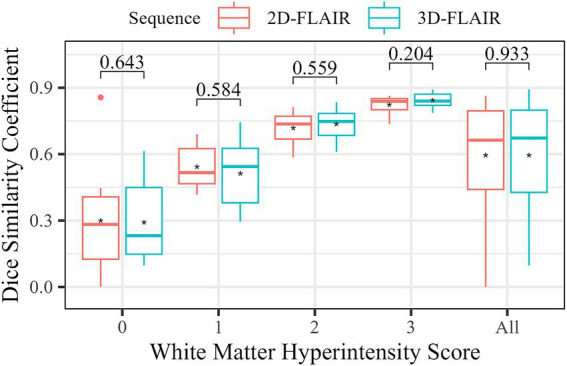
Comparison of WMH segmentation accuracy by FLAIR sequence type. The boxplot shows the distribution of DSC for each sequence type according to WMH. The asterisks (*) represent the mean DSC for each group. The numbers at the top indicate the results of statistical tests comparing the DSC differences between two groups. A Mann–Whitney U test was performed for WMH 0 and WMH all (all subjects), while a two-sample t-test was used for the other groups. A *p*-value ≥ 0.05 indicates no significant difference between the DSC in FLAIR sequence types.

The segmentation performance of Neurophet AQUA using the T2-FLAIR sequence, in relation to lesion load and T2-FLAIR sequence dimensions, is summarized in a boxplot categorized by WMH scores (Figure 5). A comparison of the DSC between 2D and 3D FLAIR sequences revealed no significant differences across all WMH score groups. Specifically, for WMH Score 0, the DSC was 0.3 ± 0.3 for 2D FLAIR and 0.3 ± 0.2 for 3D FLAIR (*p* = 0.643). For WMH Score 1, it was 0.5 ± 0.1 for 2D FLAIR and 0.5 ± 0.2 for 3D FLAIR (*p* = 0.584). For WMH Score 2, both sequences showed identical values of 0.7 ± 0.1 (*p* = 0.559). For WMH Score 3, the DSC was 0.8 ± 0.04 for 2D FLAIR and 0.8 ± 0.03 for 3D FLAIR (*p* = 0.204). Additionally, an overall comparison of DSC between 2D and 3D FLAIR, irrespective of WMH score, showed no significant differences (0.6 ± 0.2 vs. 0.6 ± 0.2, *p* = 0.933).

## 4 Discussion

This study aimed to evaluate the performance of Neurophet AQUA in three key areas: segmentation accuracy for cortical regions, repeatability and reproducibility of volume measurements, and WMH segmentation. To assess the T1-weighted MRI brain segmentation pipeline of Neurophet AQUA, we compared it to FreeSurfer (version 7.3.2, cross-sectional pipeline), a widely used tool in neuroimaging. Neurophet AQUA showed high segmentation accuracy on T1-weighted MRI, with strong agreement with FreeSurfer’s outputs. The reliability of repeated measurements was robust, with minimal variation observed across multiple segmentations. Additionally, the lesion segmentation pipeline produced consistent results from various T2-FLAIR images with the respective ground truth WMH label masks, demonstrating the tool’s versatility across different imaging sequences.

Although no statistically robust differences were found after adjusting for multiple comparisons, the comparisons revealed several important trends. Both tools performed similarly in segmenting cortical GM–WM boundaries across the four lobes (frontal, temporal, parietal, occipital), as well as the hippocampus. In the left hippocampus, AQUA received slightly higher scores (2.83 ± 0.37 vs. 2.67 ± 0.54, *p* = 0.029); however, this nominal difference did not remain significant after correction and should be interpreted cautiously.

It is worth noting that Neurophet AQUA does not require additional optimization pipelines, unlike FreeSurfer, which uses specialized pipelines for hippocampal subfield parcellation. Although FreeSurfer’s specialized pipeline may offer a more detailed segmentation of the hippocampus, the additional complexity and workflow requirements could limit its use in general neuroimaging applications. In contrast, Neurophet AQUA offers a more streamlined and user-friendly approach, making it potentially more applicable for broader neuroimaging studies. However, this study did not compare the subfield parcellations, which could provide further insights into hippocampal segmentation performance.

No significant differences were found in the segmentation of other regions such as the frontal and temporal lobes, where both tools achieved ideal ratings (grade 3, ideal) in all subjects. These findings suggest that Neurophet AQUA and FreeSurfer perform similarly in many brain regions, meaning that Neurophet AQUA may be useful for clinical applications. In hippocampal segmentation, Neurophet AQUA showed a nominal difference; however, this study should be considered a benchmark comparison rather than a superiority assessment. Future studies with larger sample sizes, more tools, and broader brain regions, along with rigorous statistical approaches including multiple comparison correction, will be necessary to draw firm conclusions about performance differences.

The scan-rescan repeatability of both Neurophet AQUA and FreeSurfer was excellent (ICC > 0.8), with Neurophet AQUA demonstrating particularly high consistency (ICC = 0.999) in ICV, which enhances its robustness in cross-sectional studies. Most brain regions, including the frontal, temporal, and parietal lobes, showed excellent repeatability (ICC ≥ 0.9). These results suggest that Neurophet AQUA may be suitable for clinical and research applications requiring high reproducibility. In addition, Neurophet AQUA demonstrated no significant volume differences across repeated measures, suggesting it may be more robust in handling scan variability.

Bland–Altman analysis supported these findings, showing that Neurophet AQUA had less bias between test and retest measurements than FreeSurfer, particularly for ICV. The narrower limits of agreement (LoA) for Neurophet AQUA indicate more consistent performance with fewer outliers, especially in the occipital lobe and hippocampus. While both tools showed similar reliability in hippocampal segmentation, Neurophet AQUA’s results were more consistent, with smaller bias and narrower LoA, especially on the right. This suggests Neurophet AQUA is suitable for clinical applications requiring stable, reproducible segmentation, such as longitudinal studies or disease monitoring.

Regarding inter-scanner reproducibility, Neurophet AQUA showed higher consistency across MRI scanners compared with FreeSurfer (average ICC: Neurophet AQUA = 0.850 vs. FreeSurfer = 0.788). AQUA also showed high consistency in ICV and brain-wide measurements (ICC = 0.983 and 0.935), whereas FreeSurfer had more variability, particularly in the occipital lobe and hippocampus. These results provide exploratory evidence that Neurophet AQUA may offer more stable performance in multi-center studies or settings using multiple scanner types. However, as the validation dataset is limited to 15 subjects (4 3 T scanners, 15 weeks), replication in a larger, multi-center cohort is required. Accordingly, the reproducibility of these findings should be verified in future studies before applying them to broader populations.

One limitation of the T1-based segmentation analysis is the use of FreeSurfer’s standard pipeline, which analyzes many brain regions in one run. This enabled comparison across regions, including the hippocampus. However, future studies may benefit from using FreeSurfer’s specialized hippocampal subfield pipeline for more targeted comparisons in neurodegenerative disease contexts. Additionally, FreeSurfer Version 8.0 (released on February 27, 2025) introduces several functional updates and deep learning–based modules, such as SynthSeg, SynthStrip, and SynthMorph, which improve cortical and subcortical segmentation accuracy and reduce processing time, albeit at higher memory requirements. Given the significant methodological changes in this version, comparative evaluation with other segmentation tools using these updated algorithms represents an important direction for future research. While this study relied on FreeSurfer’s standard pipeline for T1-weighted segmentation, future research could gain insights from systematic comparisons with other automated tools. For instance, FastSurfer provides accelerated GPU-based processing, whereas VolBrain offers automatic segmentation of T1-weighted images and detection of WMH from FLAIR images without local installation. It is relatively fast and freely accessible, but limited to 10 scans per day. Such comparative analyses could help determine the relative accuracy, efficiency, and suitability of different pipelines focused on neurodegenerative disease.

Regarding WMH segmentation on T2-FLAIR images, both 2D and 3D sequences showed similar DSC values, suggesting that Neurophet AQUA can accurately segment WMH even on 2D data. This is useful for retrospective analysis of older scans. This trend was consistently observed across a range of WMH scores, indicating reliable WMH segmentation regardless of lesion burden. The study by Lee et al. (9) validated AQUA’s WMH segmentation performance on T2-FLAIR images against existing automated and semi-automated segmentation methods (LGA, LPA, SLS, UBO, BIANCA). AQUA demonstrated higher accuracy than existing methods across diverse datasets and scanner environments. Considering these findings collectively, Neurophet AQUA supports reliable WMH segmentation even in multi-center, multi-scanner environments. However, since comparisons were not made within the same subjects, further studies are needed for equivalence assessment. Meanwhile, relatively low DSC values were observed for WMH scores of 0 (DSC mean: 2D = 0.300, 3D = 0.292), corresponding to lesion volumes below 1 mL. This aligns with previous reports indicating reduced performance for very small lesions (<0.2 mL) or multiple small lesions (34, 35). Therefore, the low DSC observed in this study can be interpreted as reflecting the general difficulty in segmenting small WMHs rather than an inherent limitation of the AQUA algorithm.

## 5 Conclusion

This study compared the segmentation performance and measurement reliability of Neurophet AQUA on T1-weighted MRI with FreeSurfer, and assessed the WMH segmentation performance of T2-FLAIR images on Neurophet AQUA. The results that T1- weighted MRI segmentation showed good segmentation quality performance for both Neurophet AQUA and FreeSurfer in most regions, while both algorithms exhibited good scan-rescan repeatability and moderate or better inter-scanner reproducibility. Notably, this comparison was performed using FreeSurfer version 7.3.2 with the standard recon-all pipeline, and the findings may not be directly generalizable to newer versions (e.g., version 8.0) that incorporate deep learning–based modules such as SynthSeg and SynthStrip. Further comparative validation using these updated frameworks will be an important direction for future research. No significant differences were observed in WMH segmentation performance between 2D and 3D T2-FLAIR sequences. These findings confirm that Neurophet AQUA provides reliable results across various MRI environments.

Neurophet AQUA is approved as a medical device by both the U.S. FDA and the Korean Ministry of Food and Drug Safety. This study highlights its potential for clinical application, offering efficient and accurate volumetric measurements in diverse clinical settings, which can support patient-specific diagnosis and treatment planning.

Future research should include comparative validation using T2-FLAIR data and further evaluate reproducibility and reliability across diverse patient cohorts. These efforts will enable a more comprehensive assessment of Neurophet AQUA’s capabilities.

In conclusion, Neurophet AQUA shows high promise as a tool for diagnosing and monitoring neurodegenerative diseases. Its utility is expected to grow with continued research and broader clinical application.

## Data Availability

The data analyzed in this study is subject to the following licenses/restrictions: the datasets used and/or analyzed during the current study are available from the corresponding author on reasonable request. Requests to access these datasets should be directed to Hyunjae Yu, yhj93@neurophet.com.

## Footnotes

1
https://github.com/TorchIO-project/torchio

2 freely available at https://surfer.nmr.mgh.harvard.edu/.

## Members of the Alzheimer’s Disease Neuroimaging Initiative

Data used in preparation of this article were obtained from the Alzheimer’s Disease Neuroimaging Initiative (ADNI) database (adni.loni.usc.edu). As such, the investigators within the ADNI contributed to the design and implementation of ADNI and/or provided data but did not participate in analysis or writing of this report. A complete listing of ADNI investigators can be found at: http://adni.loni.usc.edu/wp-content/uploads/how_to_apply/ADNI_Acknowledgement_List.pdf.
