# Supplementary material for: Multi-center evaluation of Neurophet AQUA for brain MRI segmentation: T1 compared with FreeSurfer and T2-FLAIR compared with ground truth
Source: Front Neurol. 2025 Dec 17;16:1672133. doi: 10.3389/fneur.2025.1672133 (PMC12753458; doi:10.3389/fneur.2025.1672133)
Supplement: Supplementary file 1 [file Table_1.DOCX]

Supplementary Material

# Supplementary Tables

**Supplementary Table S1.** Expert visual ratings for T1-weighted MRI segmentation quality. Expert radiologists' assessments of segmentation quality for T1-weighted MRI, categorized into cortical tissue boundaries, segmented lobes, and hippocampal segmentation.

| **Segmentation quality** | **Description** | **Scale** |
| --- | --- | --- |
| Cortical boundaries | Segmentations of cortical tissue boundaries | 1 – poor: >25% incomplete segmentation of gray matter or inclusion of non-gray matter  2 – medium: 10–25% incomplete segmentation of gray matter or partial inclusion of non-gray matter  3 – ideal: <10% incomplete segmentation of gray matter and no inclusion of non-gray matter |
| Lobar boundaries (borders between lobes and accuracy of their location) | Boundaries for cortical lobes (**Table S2**)   - Frontal (left/right) - Parietal (left/right) - Temporal (left/right) - Occipital (left/right) | 1 – poor: One gyrus is completely misclassified  2 – medium: One or more gyri are partially misclassified  3 – ideal: All gyri are correctly assigned |
| Hippocampus boundaries | Segmentation boundaries of the hippocampus (left/right) | 1 – poor: >25% non-hippocampal tissue included or hippocampal tissue not included in segmentation  2 – medium: 10–25% non-hippocampal tissue included or hippocampal tissue not included in segmentation  3 – ideal: <10% non-hippocampal tissue included or hippocampal tissue not included in segmentation |

*Abbreviation: Left, Left hemisphere; Right, Right hemisphere.*

**Supplementary Table S2.** Lobe mapping: Each lobe comprises following cortical regions of both hemispheres.

| **Lobe** | **Cortical region** |
| --- | --- |
| Frontal lobe | - Caudal middle frontal - Lateral orbito frontal - Medial orbito frontal - Pars opercularis - Pars orbitalis - Pars triangularis - Precentral - Rostral middle frontal - Superior frontal - Frontal pole - Paracentral |
| Parietal lobe | - Inferior parietal - Postcentral - Precuneus - Superior parietal - Supramarginal |
| Temporal lobe | - Bankssts (bank of the posterior superior temporal sulcus) - Entorhinal - Fusiform - Inferior temporal - Middle temporal - Parahippocampal - Superior temporal - Temporal pole - Transverse temporal |
| Occipital lobe | - Cuneus - Lateral occipital - Lingual - Pericalcarine |

**Supplementary Table S3.** Bland-Altman analysis for scan-rescan repeatability.

|  | | **Bias (95% LoA)** | |
| --- | --- | --- | --- |
|  | | **Neurophet AQUA** | **FreeSurfer** |
| **ICV** | | -0.089 (-9.600, 9.422) | 9.364 (-29.044, 47.773) |
| **Whole Brain** | | 0.824 (-17.418, 19.066) | -2.567 (-39.406, 34.272) |
| **Frontal Lobe** | **Left** | -0.357 (-5.572, 4.858) | 0.069 (-4.291, 4.428) |
|  | **Right** | -0.424 (-6.471, 5.624) | -0.240 (-5.211, 4.731) |
| **Temporal Lobe** | **Left** | -0.420 (-3.847, 3.006) | 0.528 (-4.342, 5.398) |
|  | **Right** | -0.302 (-4.156, 3.552) | 0.473 (-4.537, 5.483) |
| **Parietal Lobe** | **Left** | -0.268 (-4.359, 3.823) | 0.013 (-4.040, 4.066) |
|  | **Right** | -0.327 (-4.235, 3.581) | 0.186 (-5.005, 5.377) |
| **Occipital Lobe** | **Left** | -0.132 (-3.413, 3.149) | 0.053 (-3.192, 3.297) |
|  | **Right** | -0.350 (-4.235, 3.535) | 0.227 (-4.588, 5.042) |
| **Hippocampus** | **Left** | -0.010 (-0.426, 0.406) | 0.025 (-0.409, 0.460) |
|  | **Right** | 0.023 (-0.450, 0.496) | -0.014 (-0.264, 0.236) |

*Bias is the average of the measurement differences in scan-rescan. Volume measurements for each region were in cc.
Abbreviation: LoA, Limits of agreement; ICV, intracranial volume; Left, Left hemisphere; Right, Right hemisphere.*

**Supplementary Table S4.** Limits of agreement with the mean for inter-scanner reproducibility. The inter-scanner reproducibility of Neurophet AQUA and FreeSurfer was evaluated by measuring the volumes of T1-weighted MRI scans in 15 patients on four types of scanners Siemens Trio, Siemens Prisma, GE Discovery 750, Philips Achieva) to compare the limits of agreement with the mean.

|  | | **LOAM** | |
| --- | --- | --- | --- |
|  | | **Neurophet AQUA** | **FreeSurfer** |
| ICV | | 27.995 | 97.580 |
| Whole Brain | | 41.207 | 54.430 |
| Frontal Lobe | Left | 5.815 | 5.470 |
|  | Right | 6.064 | 8.368 |
| Temporal Lobe | Left | 2.491 | 3.228 |
|  | Right | 2.380 | 3.926 |
| Parietal Lobe | Left | 3.807 | 4.306 |
|  | Right | 4.570 | 5.163 |
| Occipital Lobe | Left | 2.117 | 2.041 |
|  | Right | 2.000 | 3.729 |
| Hippocampus | Left | 0.286 | 0.342 |
|  | Right | 0.774 | 0.341 |

*Abbreviation: LOAM, Limits of agreement with the mean; ICV, intracranial volume; Left, Left hemisphere; Right, Right hemisphere.*

# Supplementary Figures

**
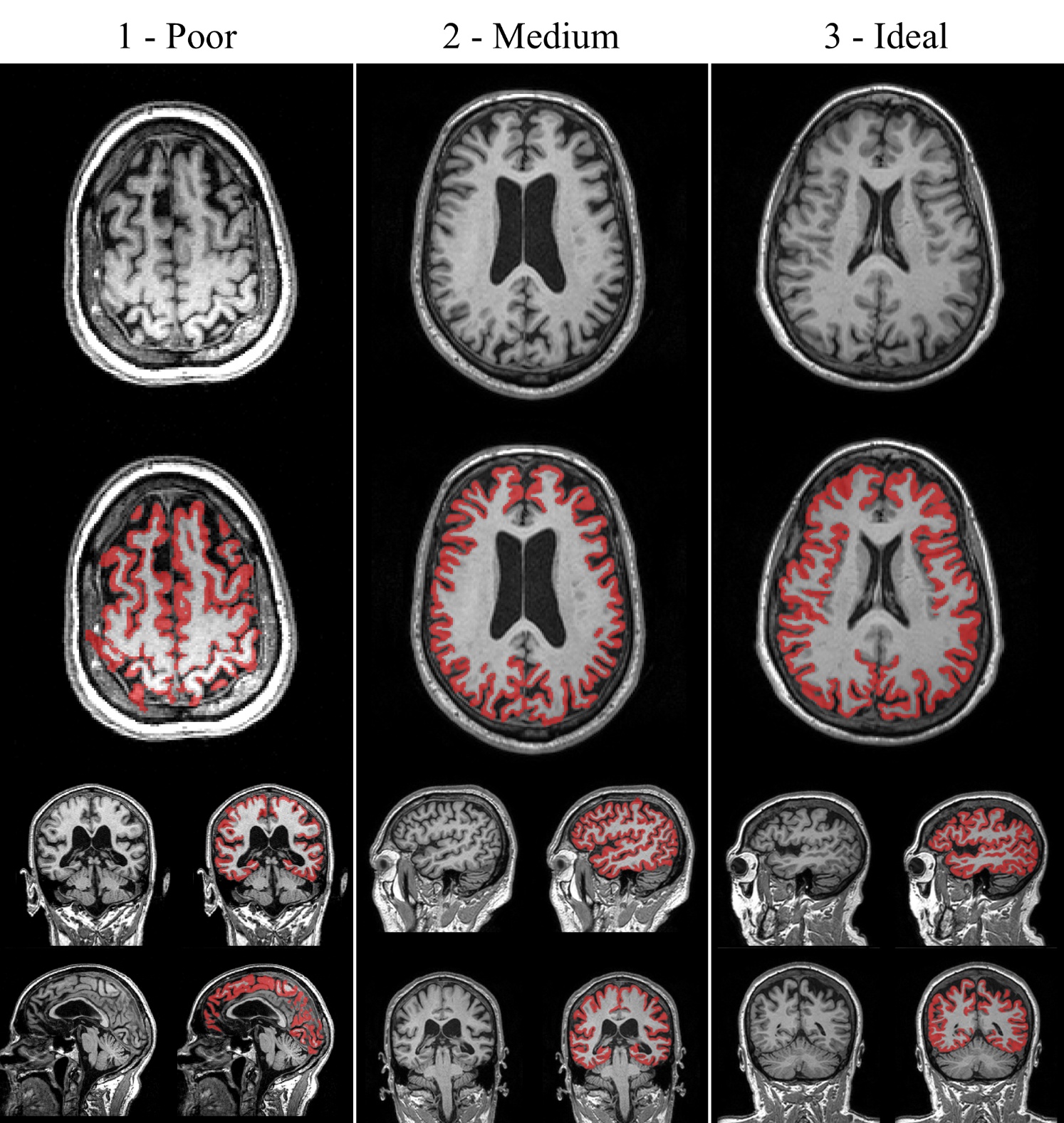
**

**Supplementary Figure S1.** Example images showing segmentation quality of cortical boundaries across three grading levels.


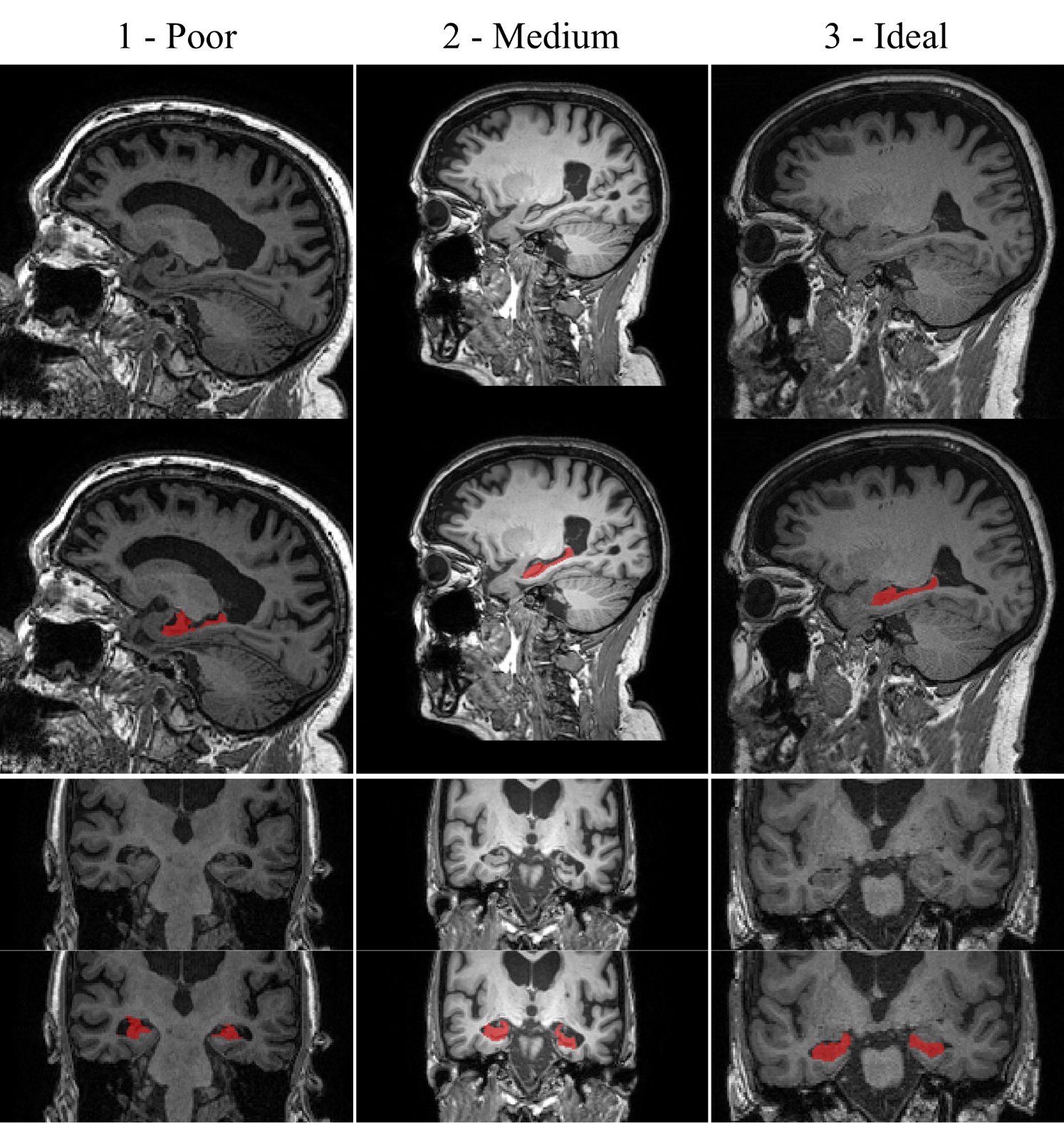


**Supplementary Figure S2.** Example images showing segmentation quality of hippocampal across three grading levels.

**
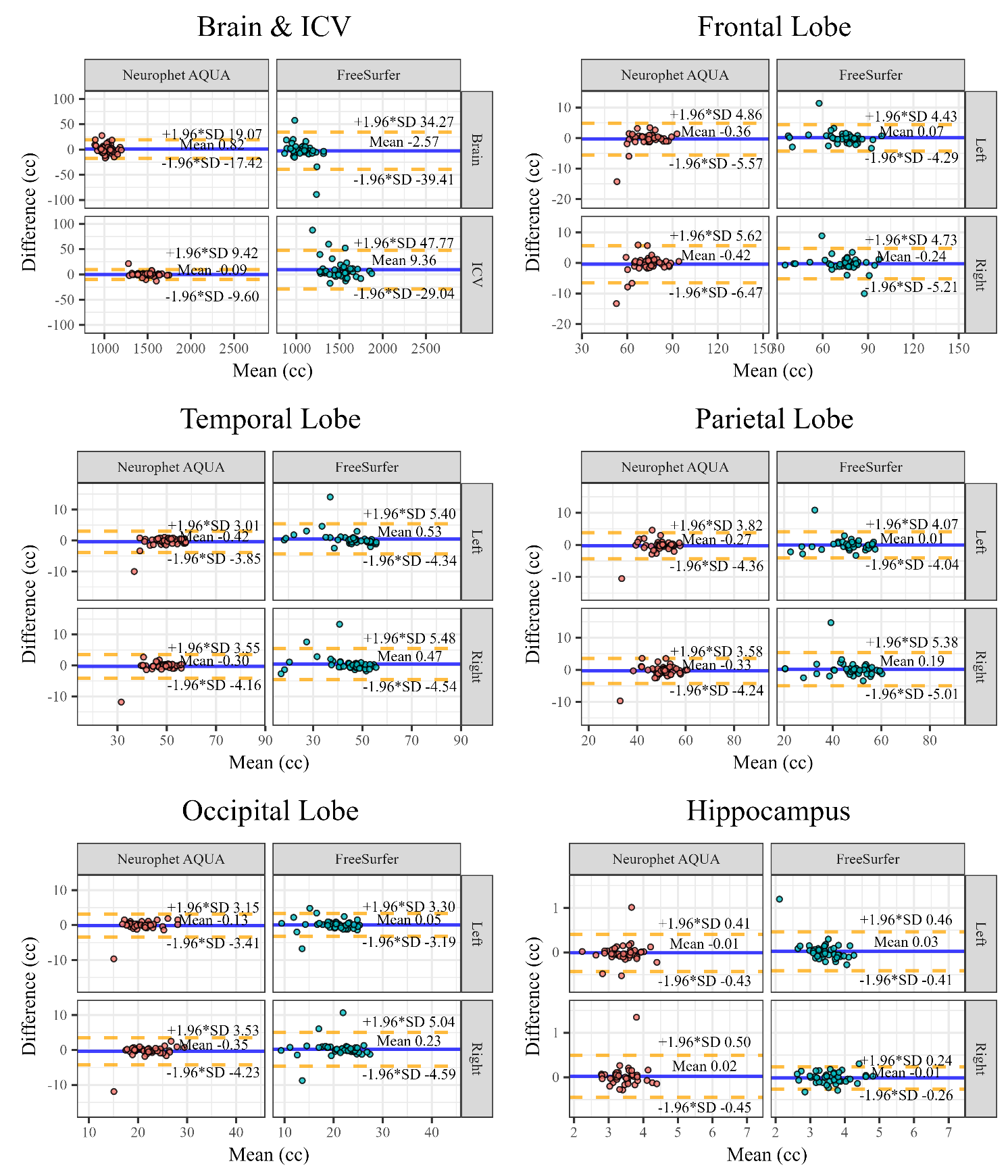
**

**Supplementary Figure S3.** Bland-Altman plots of scan-rescan measurements at Neurophet AQUA and FreeSurfer. X-axis: mean volume between the scan and rescan. The unit of volume on the X-axis is cc. Y-axis: volume difference between the scan and rescan. The unit of volume on the Y-axis is cc. The mean of difference is indicated by blue line. The lower (-1.96 SD) and upper (+1.96 SD) limits of agreement are indicated by dotted yellow lines. A negative difference on the y-axis indicates that the volume measured in retest image was smaller than volume measured in test image.

Abbreviation: ICV, intracranial volume; Left, left hemisphere; Right, right hemisphere; SD, Standard Deviation.

**
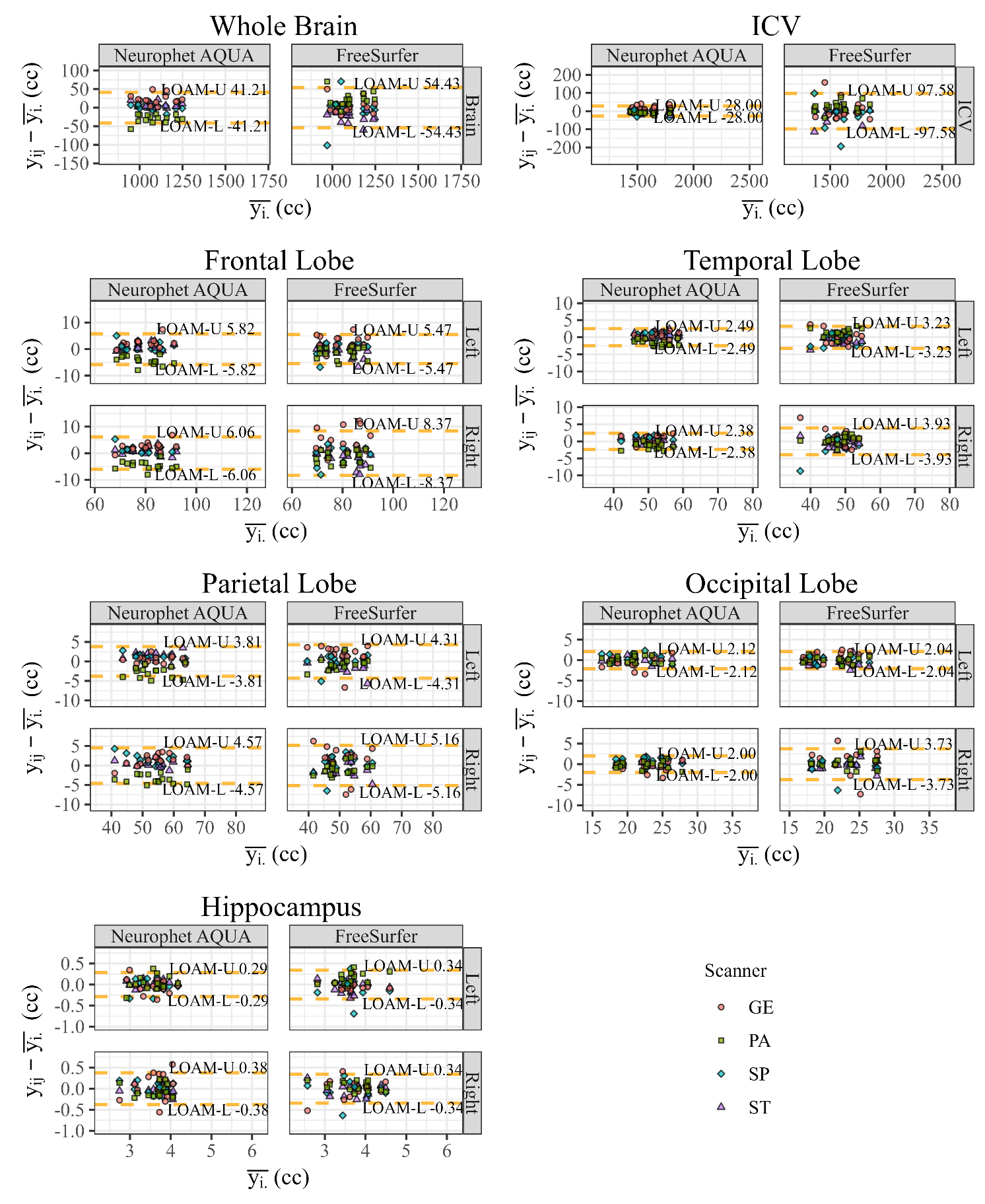
 Supplementary Figure S4.** Limits of agreement with the mean in the inter-scanner reproducibility. X-axis: mean volume of images taken from four types of scanners. The unit of volume on the X-axis is cc. Y-axis: difference between the mean volume of an individual subject and the volume per scanner. The unit of volume on the Y-axis is cc. The lower and upper limits of agreement with the mean are indicated by dotted yellow lines.

Abbreviation: LOAM-L, lower limits of agreement with the mean; LOAM-U, upper limits of agreement with the mean; ICV, intracranial volume; Left, left hemisphere; Right, right hemisphere; GE, GE Discovery 750; PA, Philips Achieva; SP, Siemens Prisma; ST, Siemens Trio.
